# Editorial: The role of immunity and inflammation in aging and aging-related diseases

**DOI:** 10.3389/fmolb.2026.1866979

**Published:** 2026-05-19

**Authors:** Luca Soraci, Liangliang Dai

**Affiliations:** 1 Unit of Geriatric Medicine, Italian National Research Center on Aging (INRCA IRCCS), Cosenza, Italy; 2 Northwestern Polytechnical University, Xi’an, China

**Keywords:** aging, immune system, inflammaging, inflammation, older

Aging is a biological process characterized by a progressive deterioration of multiple physiological organs and systems with subsequent increased individual vulnerability and susceptibility to developing acute and chronic diseases. One of the main contributors to aging is represented by inflammaging, a state of chronic, low-grade sterile inflammation, which may contribute to the pathogenesis of a vast number of age-related diseases and syndromes, including cardiovascular diseases, neurodegenerative disorders, metabolic disorders, and cancer (Franceschi et al., 2000; Franceschi and Campisi, 2014). More specifically, inflammaging is characterized by a dysregulation of both innate and adaptive immune responses that creates a persistent pro-inflammatory environment that affects host defense and promotes tissue damage (Franceschi and Campisi, 2014). Understanding the molecular and cellular roots of age-related immune dysfunction underlying the onset of specific aging-related diseases is one of the most important challenges of biomedical research.

Four original investigations recently published have depicted different but interconnected dimensions of immunity and inflammation in aging.

Jakobsche-Policht et al. addressed the complex interplay between platelet biology, hemostasis, and vascular inflammation in atherosclerosis, which is very common in older patients. Their study provided a comprehensive analysis of the protease-activated receptor 1 (PAR-1), a G-protein coupled receptor expressed on platelets and platelet derived microparticles (PMPs), in two clinically distinct forms of macrovascular diseases: the atherosclerosis obliterans (AO) and diabetic macroangiopathy (DM). By using a complex approach integrating flow cytometry, RT-PCR, aggregometry, and multidimensional logistic regression, the authors demonstrated that DM patients were characterized by increased TRAP-stimulated PAR-1 activation compared with AO patients and healthy controls; this may suggest that the PAR-1/thrombin axis represents a key driver of platelet hyperactivation in diabetes-accelerated atherosclerosis. Interestingly, the authors identified the −506 I/D and IVSn-14 A/T polymorphisms of the PAR-1 gene as important modulators of the susceptibility to the disease: the −506 I allele may exert a protective effect by attenuating platelet responsiveness, while the D/D and IVS-14 A/A genotypes, which favor stronger PAR-1 activation, are enriched in the DM group. Further information derived from interaction analyses: at high platelet-derived microparticles (PMP) percentages, an increase in PAR-1 activation significantly increases the odds of AO, underlining a non-linear relationship between PMP biology and vascular risk. These findings suggest that PAR-1 and PMP profiling may represent promising tools to stratify the severity of atherosclerosis; furthermore, targeting the PAR-1/thrombin interaction may hold therapeutic potential in the context of diabetic vascular disease.

Wang et al. investigated the role of neddylation in laryngeal squamous cell carcinoma (LSCC), whose incidence increases with age and is associated with significant morbidity. Neddylation represents a post-translational protein modification involving the covalent attachment of the ubiquitin-like molecule NEDD8; drawing on transcriptomic data from The Cancer Genome Atlas database and integrating them with neddylation-related gene sets from the Reactome database, the authors finally identified 79 differentially expressed neddylation-related genes (NRGs) in LSCC and construct a three-gene prognostic risk signature comprising *COMMD2*, *WSB2*, and *CUL9*. This signature was able to stratify patients into high- and low-risk groups for 3- and 5-year overall survival, with area under the receiver operating characteristic curve (AUC) values up to 0.848 in the validation set. Immune microenvironment analysis, performed using single-sample gene set enrichment analysis, revealed that WSB2 expression was negatively correlated with infiltration of activated CD8^+^ T cells, while CUL9 positively associates with activated B cells; these findings suggest that neddylation-related genes may modulate the prognosis of LSCC, partly by acting on the recruitment and functioning of immune cells. Furthermore, the study experimentally demonstrated that silencing WSB2 in LSCC cell lines substantially inhibited cell proliferation, migration, and invasion, validating its oncogenic role and identifying it as a candidate therapeutic target.

Chang et al. reported the first proteomic characterization of a multi-generation Chinese family affected by Lynch syndrome, the most common hereditary colorectal cancer syndrome, carrying a rare large genomic rearrangement in the form of a heterozygous deletion of exon 13 in the *MLH1* gene (MLH1-EX13 Del). The authors applied data-independent acquisition-based quantitative proteomics to four paired tumor and adjacent normal tissue specimens from affected carriers. This unbiased proteomic profiling revealed that MLH1-deficient colorectal tumors are characterized by a dual molecular hallmark: a) systemic suppression of oxidative phosphorylation across all five mitochondrial respiratory chain complexes, consistent with a shift toward the Warburg effect; b) robust upregulation of ribosome biogenesis proteins and a pronounced inflammatory-immune signature dominated by neutrophil infiltration markers including S100A8/A9, myeloperoxidase, and ELANE. The concurrent upregulation of antigen-presentation machinery including HLA-DRA, ITGAM, and C1QC is consistent with the well-established “hot tumor” phenotype of mismatch repair-deficient cancers and provides a molecular rationale for their favorable response to immune checkpoint inhibitors. By linking a specific germline structural variant to a coherent downstream proteomic phenotype in a familial context, this work exemplified the power of integrative multi-omics approaches for precision oncology.

Chen et al. turned the spotlight toward chronic rhinosinusitis with nasal polyps (CRSwNP), a prevalent and therapeutically challenging inflammatory airway disease in which immune dysregulation plays a central pathogenic role. Leveraging two independent Gene Expression Omnibus datasets—GSE136825 and GSE179625 — and a curated panel of 338 anoikis-related genes, patients were stratified in two distinct subtypes based on the activity of anoikis pathways. Anoikis, the programmed cell death triggered by loss of extracellular matrix attachment, is recognized as a regulator of immune homeostasis, and its dysregulation has been found to play a role in CRSwNP. Immune cell infiltration analysis using the CIBERSORT algorithm revealed that the two subtypes were characterized by distinct immune microenvironments: cluster one was characterized by overexpression of the androgen receptor (AR); cluster two was instead dominated by the parathyroid hormone-like hormone (PTHLH). A diagnostic nomogram made up of four genes, including AR, PTHLH, cadherin-3, and programmed cell death 4, achieved high diagnostic accuracy (AUC >0.90). Network analysis further identified 862 candidate drugs or compounds targeting AR, highlighting the translational potential of this molecular subtyping framework.

These works not only advance our mechanistic understanding of specific diseases but also chart a broader research agenda for the field of immunogerontology, one in which the boundaries between inflammation, immunity, metabolism, and aging are increasingly recognized as fluid, interdependent, and therapeutically exploitable.
